# Foot-to-Forearm Tactile Feedback for Lower-Limb Exoskeleton Control: A Pilot Benchmarking Study in Healthy Adults

**DOI:** 10.3390/s25227050

**Published:** 2025-11-18

**Authors:** Mafalda Aguiar, Carla Pais-Vieira, Demétrio Matos, André Perrotta, Peter Kreynin, Miguel Pais-Vieira

**Affiliations:** 1 Department of Physics, University of Aveiro, 3810-193 Aveiro, Portugal; mafaldaaguiar@ua.pt; 2 Center for Interdisciplinary Research in Health (CIIS), Faculty of Health Sciences and Nursing, Portuguese Catholic University, 4169-005 Porto, Portugal; cvieira@ucp.pt; 3 Research Institute for Design, Media and Culture, Polytechnic Institute of Cávado and Ave, 4750-299 Barcelos, Portugal; dmatos@ipca.pt; 4 Centre for Informatics and Systems of the University of Coimbra (CISUC), Department of Informatics Engineering, University of Coimbra, 3030-290 Coimbra, Portugal; avperrotta@dei.uc.pt; 5 ExoAtlet, 4354 Esch-sur-Alzette, Luxembourg; p.kreynin@exoatlet.com; 6 Institute of Biomedicine (iBiMED), Department of Medical Sciences, University of Aveiro, 3810-193 Aveiro, Portugal

**Keywords:** exoskeleton, tactile feedback, rehabilitation, gait parameters

## Abstract

Real-time tactile feedback has been applied in exoskeleton-based neurorehabilitation, particularly in spinal cord injury, through Foot-to-Forearm Feedback (FFF) at ground contact. Its effects on healthy individuals across varied terrains remain less understood. This pilot study investigated seven healthy participants controlling a lower-limb exoskeleton under two conditions (with and without FFF) across five scenarios: Flat, M-Shape, A-Shape, V-Shape, and Random. Objective measures included step count, sacroiliac joint (SIJ) segment length, and SIJ angle. Subjective experience was assessed by overall preference and a Global Experience Index (GEI) derived from questionnaire ratings. Analyses showed no consistent differences in step count, SIJ length, or SIJ angle between Control and FFF. In three runs, technical issues occurred (e.g., a loose cable). Subjective data showed that five of seven participants (71.4%) preferred FFF, one reported no difference, and one preferred the Control condition. Scenario-specific analyses indicated a preference for FFF in Flat (5/7 = 71.4%), but a lower preference in Random (2/6 = 33.3%). GEI scores decreased under A-Shape and Random conditions, though FFF partially mitigated the reduction in A-Shape. These preliminary findings suggest that FFF may influence subjective experience in healthy users, but confirmation will require larger samples and further investigation across scenarios.

## 1. Introduction

Lower limb exoskeletons combined with tactile feedback are increasingly used in neurorehabilitation settings [1,2]. Exoskeleton-assisted standing and walking have been reported to improve outcomes in patients with spinal cord injury (SCI), leading to increased bone density and lean body mass, reduced neuropathic and nociceptive pain, and improved cardiovascular and bowel function [3]. Such effects suggest that an exoskeleton is not merely a support device but may also serve as a therapeutic adjuvant in SCI rehabilitation [3].

Despite these advances, walking with an exoskeleton alone has not consistently improved neurological function below the level of injury, such as somatosensory perception or voluntary motor control [4]. However, when combined with tactile feedback—or when exoskeletons are controlled directly by brain activity—evidence indicates increased neuroplasticity and partial neurological recovery, even in individuals with clinically complete SCI [5].

The mechanisms underlying these effects are not fully understood, but embodiment is considered a key factor [6,7]. Embodiment refers to how the brain represents the body and how this representation can be modified by user experience and external conditions [8,9,10]. Multi-modal feedback (tactile, thermal, auditory, visual, and vestibular) can enhance embodiment [5,6,7], and recent reports suggest that the combination of embodiment, feedback, and exoskeleton use contributes to beneficial neuroplastic changes in SCI rehabilitation. Moreover, variations in virtual terrains (e.g., rural vs. urban environments, flat vs. inclined surfaces) have been shown to influence embodiment and perceived effort [6,7]. These findings highlight the importance of studying how testing scenarios and feedback modalities shape user experience.

A specific form of tactile stimulation, Foot-to-Forearm feedback (FFF), has been used to trigger neuroplastic changes in SCI rehabilitation [5]. In this approach, a tactile stimulus is delivered to the forearm when the exoskeleton foot contacts the ground. While this strategy has shown promise in rehabilitation, it is inherently non-physiological: the forearm receives sensory input representing the sole of the foot. In healthy individuals, such additional input may not always be beneficial. FFF could even interfere with exoskeleton control, as it requires users to process an extra, mismatched signal. Nevertheless, previous studies [6,7], as well as preliminary findings from our group, showed that both SCI patients and healthy control participants reported preferring FFF in virtual reality (i.e., when the avatar’s foot contacted the ground, a forearm actuator vibrated). This feedback reduced the need to constantly monitor the ground, as participants could sense foot contact through vibration. Studying healthy individuals thus provides a controlled baseline to assess whether additional vibrotactile input supports or interferes with exoskeleton use, which is essential before translating such approaches to clinical populations.

Prior research has shown that exoskeleton walking across different terrains elicits distinct neural activity patterns depending on individual characteristics [11]. It remains unclear whether FFF serves as a helpful aid or a disruptive input, particularly in environments of varying difficulty. This raises a key question: can FFF support exoskeleton use across terrains, or does it add unnecessary complexity?

To begin addressing this question, we conducted a pilot study involving seven healthy participants. The aim was not to test rehabilitation outcomes but rather to describe whether FFF affects exoskeleton control or user experience in different terrain conditions. Such exploratory work can clarify whether FFF serves merely as a comfort-enhancing feature, as an effective aid, or as a potential obstacle for healthy users. As others have emphasized [1], studying the user’s experience during exoskeleton control with and without FFF is essential for the translation of these findings to SCI rehabilitation. Ultimately, these first insights will contribute to understanding the interplay among embodiment, user experience, and neuroplastic potential, and help establish benchmarking protocols that enable broader generalization of exoskeleton applications [12,13].

The objective of this pilot study was to describe the user experience in a small sample of healthy participants operating an exoskeleton with and without Foot-to-Forearm tactile feedback in different scenarios. These scenarios are characterized by increases and decreases in the transverse and longitudinal axes of a platform, such that different levels of difficulty are present during crossing of a platform.

## 2. Materials and Methods

**Figure 1:**
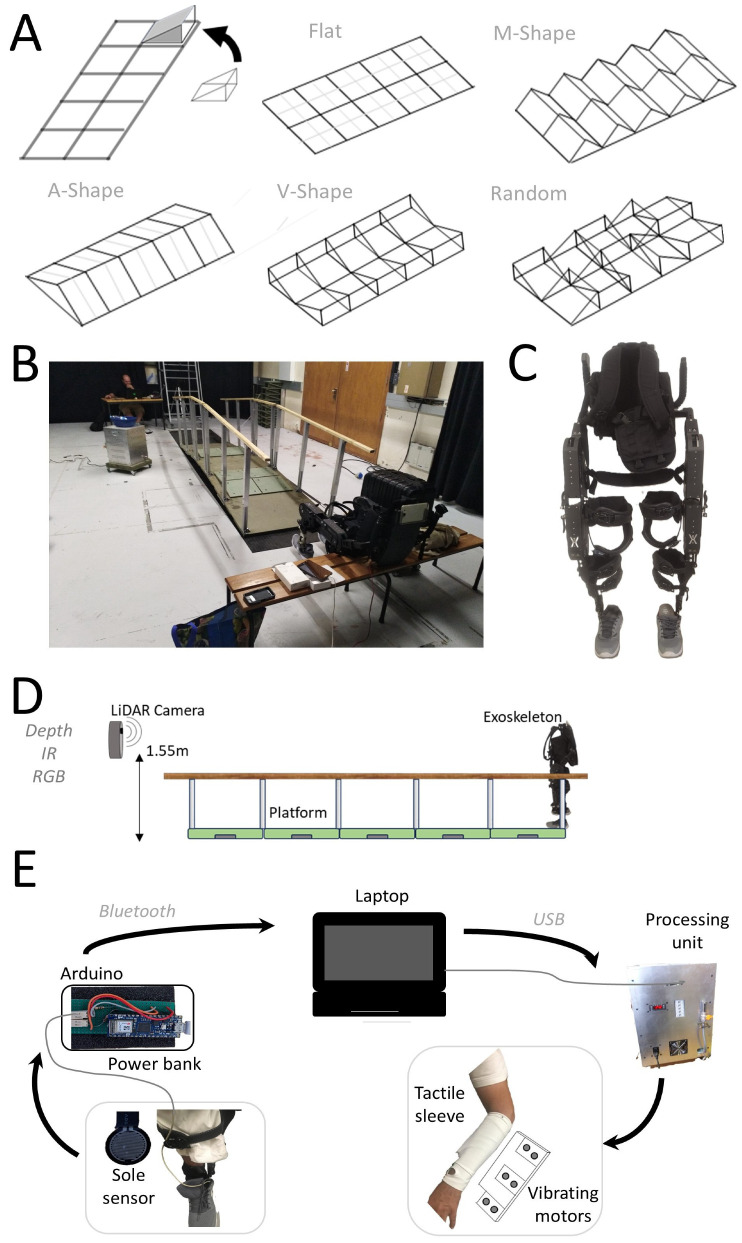
Experimental setup. (**A**) Different scenarios used. On the top left is presented the platform in the Flat scenario configuration. Modular blocks allow the generation of different scenarios. (**B**) Example showing the adjustable catwalk with movable panels and parallel bars. This specific scenario was not used in the experiments. To change between scenarios, the small movable ramp units were removed and the large lower panels were lowered to the ground. (**C**) The ExoAtlet^®^ exoskeleton. (**D**) Description of the LiDAR system used. (**E**) Description of the Foot-to-Forearm Feedback (FFF).

In the present study participants were tested in a catwalk while using an exoskeleton (Control condition) or using an exoskeleton with Foot-to-Forearm feedback (FFF condition). The platform with the catwalk could be changed to allow for scenarios with different configurations to be tested (Figure 1).

### 2.1. Participants

Before the video recordings began, the experimental procedure was thoroughly explained to all participants, and any questions were addressed. All participants then signed an informed consent form and agreed that their image could be used. The study was approved by the Ethics Committee for Health Sciences of the Universidade Católica Portuguesa (approval number 99/2022, Porto, Portugal).

Experiments were conducted at the Universidade Católica Portuguesa (Porto, Portugal) between August and September 2022, with a total of seven participants (three females and four males). Inclusion criteria were height between 1.50 and 1.90 m, body weight below 100 kg, and the ability to support the exoskeleton’s weight without pain. Exclusion criteria were age under 18 years or the presence of physical impairments. Participants visited the university once and completed the session in a single visit. No familiarization sessions with the exoskeleton were conducted. Six of the seven participants had prior experience with the exoskeleton; the one without prior experience was not the participant who later reported discomfort with the tactile feedback. Sessions were conducted in a video recording studio, where environmental conditions were maintained at 24–26 °C and 50–60% relative humidity.

### 2.2. Materials

This study used a platform, an exoskeleton and a Foot-to-Forearm Feedback (FFF) device. The platform was developed in the context of the Eurobench Robotic Framework for Bipedal Locomotion Benchmarking (described below). The ExoAtlet^®^ exoskeleton and the Foot-to-Forearm Feedback gear were used in both the Control condition and the FFF condition. In the FFF condition, the Foot-to-Forearm Feedback was turned on (described below).

### 2.3. Platform

The platform used for participant testing is shown in Figure 1. It consists of a 5 m long structure composed of five 1 m panels equipped with adjustable lateral bars. Figure 1 illustrates the platform’s capabilities. In this image, the central panel has been raised, and eight additional ramp units (i.e., smaller panels) with a 5-degree slope have been added. This particular configuration was not used in the experiments and is included here solely to demonstrate the flexibility of the setup. Various types of ramp units (e.g., with different slopes) can be used to adjust the task difficulty (Figure 1A).

In the present study, all ramp units had a 5-degree slope. We have determined in preliminary experiments that this was a moderate difficulty slope and therefore suitable for testing the different scenario configurations.

As shown in Figure 1A, five different scenarios were tested here: Flat (no additional ramp units), A-Shape (ramp units aligned to increase the platform’s longitudinal axis), V- Shape (ramp units aligned to decrease the longitudinal axis), M-Shape (ramp units partially aligned to alternately increase and then decrease the transverse axis), and Random (ramp units positioned to avoid alignment along either axis). These different scenarios were chosen because they were expected to generate moderate levels of difficulty in the control of the exoskeleton, therefore allowing us to test the effect of FFF.

### 2.4. Exoskeleton

The ExoAtlet^®^ exoskeleton was used in all experiments, as previously described [14]. The ExoAtlet^®^ is a lower-limb exoskeleton designed to support early locomotion in patients with neurological disorders. It consists of a metallic frame with electric motors, mechanical actuators, and an onboard computer. Smart crutches are included with the system to enhance control and safety; however, in this study, they were not used, as these functions were provided by the parallel bars of the walking platform. Each hip and knee joint is powered by two motors per side, providing two degrees of freedom.

The exoskeleton was operated remotely via tablet. The device also has handles located at the back to allow a support person to help control it and ensure safe equipment usage. All experiments were conducted with an experimenter behind the exoskeleton to ensure the safety of participants, as previously described [14]. The exoskeleton was controlled through one of the original algorithms included in the exoskeleton software, which consists of forward leg movements at 1 Hz.

### 2.5. Foot-to-Forearm Feedback

Foot-to-Forearm Feedback (FFF) required acquisition of force values and generation of vibrating patterns. We previously described an apparatus for tactile stimulation [6,7,15,16] which was used to deliver the tactile feedback. Here, we have combined this previous mode of delivery with an additional Arduino-based device that enabled acquisition of force data. An analog resistive sensor (Adafruit Industries, LLC, New York City, NY, USA) was connected to an Arduino, which was powered through a portable power bank (Redmi 10,000 mAh, Xiaomi, Beijing, China). The Arduino was programmed to transmit the analog force readings via Bluetooth from the sensor to a laptop controlling the tactile processing unit. The tactile processing unit then controlled the tactile stimuli delivered to the user’s forearm. Two tactile feedback sleeves were used throughout the experiment, as previously described [6,7,15,16]. Each sleeve was worn on the forearm and contained six vibrating actuators. The tactile feedback sleeve vibrated whenever the participant’s foot touched the ground, delivering FFF (Figure 1D).

### 2.6. Recordings

A LiDAR Intel RealSense L515 camera containing 3 streams (depth, IR, RGB) recording at 30 frames per second was positioned at 1.55 m from the ground. The camera resolution is 1280 × 720 pixels with a 70° horizontal and 55° vertical aperture angle. This configuration yields an effective spatial resolution of approximately ±5.4 mm horizontally and ±5.75 mm vertically at a distance of 5 m, improving as the subject moves closer to the camera. The depth resolution is <14 mm at 9 m and improves to ≈5 mm at 1 m. This camera was calibrated and used to track the distance. The Openpose Python API, V1.7.1, 2021, Python Software Foundation, Wilmington, DL, USA, was used for calculating 2D coordinates for each joint [17]. This was combined with the pixel depth information of the LiDAR camera to compose the 3D coordinates of the joints. The positions of the sacrum—representing the approximate center of mass—and of the sacroiliac joint parameters were extracted for each scenario and condition.

**Figure 2:**
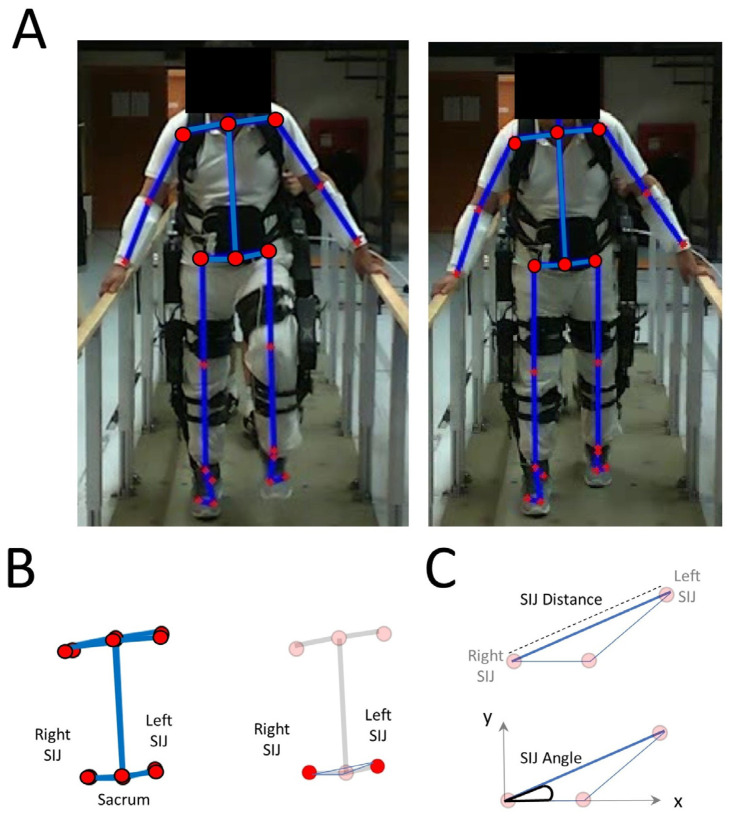
Sacroiliac joint detection and analysis. (**A**) Video capture of the different joints. (**B**) Right and left sacroiliac joints (SIJs) and the sacrum. (**C**) Segment representing the distance and angle between the right and left SIJs.

The 3D coordinates were used to calculate two geometric features: (1) the length of the segment between the right and left sacroiliac joints, and (2) the orientation of this segment relative to the coordinate axes (Figure 2). These two variables were chosen because, despite the partial restriction of lower limb movement due to the use of the exoskeleton, participants can still have control over the X and Y coordinates of where their feet will land during gait. Such control is often present in the sacroiliac joint as well as in the shoulder girdle.

The sacroiliac joint (SIJ) segment length was obtained as the Euclidean distance between the right and left sacroiliac joints based on their X and Y coordinates, and the median across all frames in a run was used for analysis. The segment angle was computed using MATLAB ’s atan2 function, R2025a, Natick, MA, USA, which calculates the orientation of a vector in the XY plane while preserving the correct quadrant and avoiding division errors inherent in a simple inverse tangent calculation. For runs with technical problems, SIJ length and SIJ angle were analyzed only during periods unaffected by the issue. Including or excluding these runs did not alter the results.

### 2.7. Safety

The exoskeleton was used in a preprogrammed mode where continuous moving forward footsteps occurred at 1 Hz. To ensure safety throughout the whole session, an experimenter was positioned behind the participant at a distance of less than one meter. Participants held the side parallel bars (Figure 2A). Gait initiation and termination were controlled by the experimenter using a notepad wirelessly connected to the exoskeleton. If a cable became loose at any point, the trial was immediately paused, the cable was resecured, and the session was subsequently resumed.

### 2.8. Session Structure

**Figure 3:**
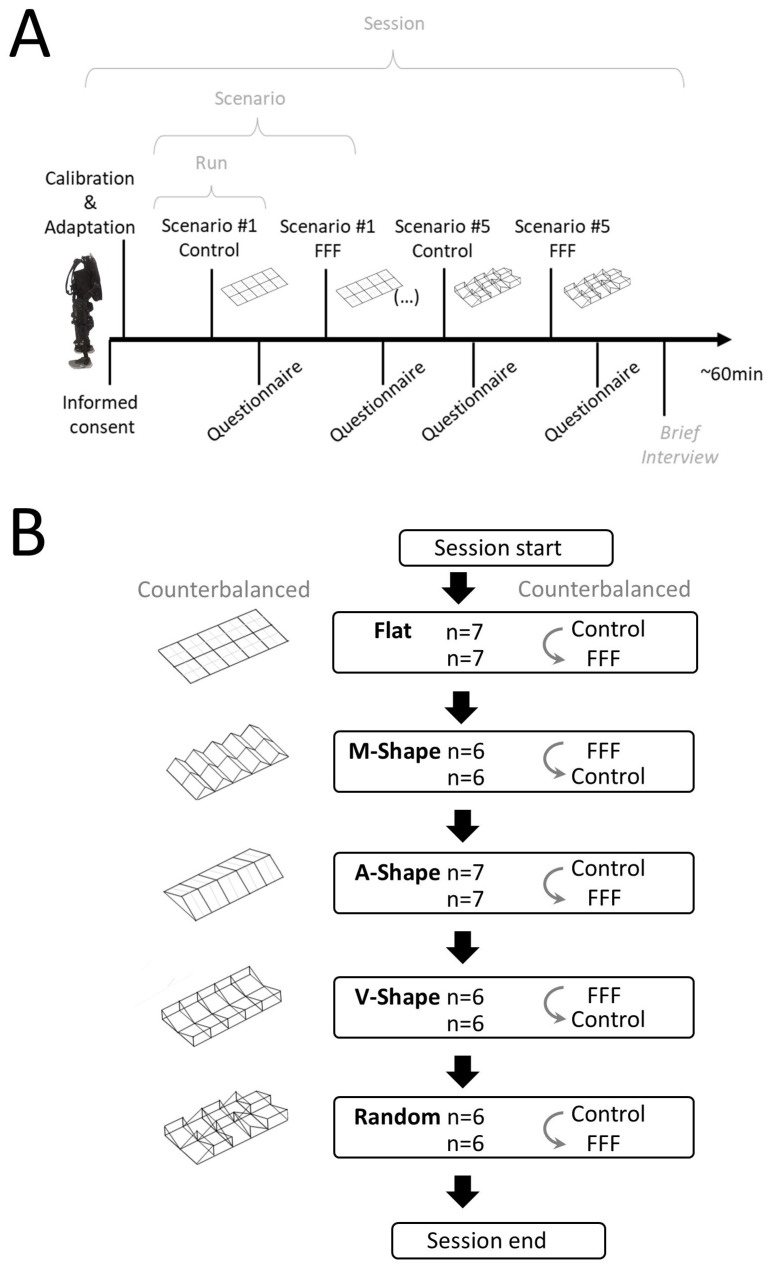
Experimental design. (**A**) Each session included a period of calibration and adaptation followed by testing in different scenarios. In each scenario, the participant would perform two runs (one in the Control condition and one in the FFF condition). (**B**) Different numbers of participants were tested in each scenario. A total of 6 participants completed all the scenarios. One participant completed only the Flat and A-Shape scenarios.

The experimental design is described in Figure 3A,B. Each session included a period of preparation characterized by calibration and adaptation, followed by a period for testing. The preparation included signing the informed consent, followed by measurement of relevant anatomical structures and then by donning the exoskeleton and the Foot-to-Forearm Feedback gear. This was performed in the sitting position. Participants then moved to an upright position, and a brief period of adaptation to the exoskeleton and the additional gear (tactile sleeve, power bank, etc.) was allowed. When participants indicated being prepared, testing began.

During testing, participants moved forward with the exoskeleton in five different scenarios with and without the FFF (i.e., ten different runs). The order of the scenarios, as well as the order of FFF use, was counterbalanced across participants. After each run (i.e., moving through the scenario with or without FFF), a questionnaire with eight items to be scored with a Likert scale (1–7) was applied.

### 2.9. Global Experience Questionnaire and Brief Interview

The questionnaire used in this study [18] included a total of eight different items referring to the use of the exoskeleton with or without the FFF. The first four items related to positive aspects of user experience: Usability, Acceptability, Perceptibility, Functionality. The last four were associated with deteriorated user experience: Stress, Fatigue, Energy Expenditure, and Attention (Table 1).

At the end of each session (Control and FFF), participants were asked whether they preferred using the exoskeleton with or without the FFF. They were also invited to provide any additional comments they considered relevant.

### 2.10. Experimental Protocol

After verifying that all prerequisites were met to start the experiment (the consent form was signed and the exoskeleton and tablet were fully charged), the exoskeleton was adjusted to fit the participant’s measurements. Lastly, each participant moved from a sitting position to a standing position, and all equipment was adjusted accordingly (i.e., moving from sitting to standing often requires adjustments to the safety straps). The participant was then allowed to adapt to the exoskeleton’s weight. Testing began once all conditions were met.

Participants were asked to walk in a straight line, starting from the beginning of the platform until the end of it, with an experimenter ensuring the safety of the exoskeleton operation from behind. For each scenario, participants performed two runs, one with the tactile feedback sleeves (FFF condition) and another without the tactile feedback (Control condition). Each condition was counterbalanced across participants.

## 3. Numerical and Descriptive Quantitative Analysis

Given the pilot nature of the present study and due to the small number of participants tested in multiple conditions, the description will consist of a quantitative and qualitative description of the results. The number of steps was counted for each run and compared. The experimenter counting the steps in each run was blind to the condition of the participant (i.e., Control or FFF). The median values for the angles between the sacroiliac joints and the sacrum, and the median values for the length of the segment between the left and right sacroiliac joints were compared. Pearson correlations were calculated for the analysis of associations between objective and subjective measures of performance, namely for the values of the GEI calculated from the questionnaire and the SIJ segment angle.

The results from the questionnaires will be presented as the mean and standard error of the mean (SEM).

Two main analyses will be performed on the questionnaires. The first will consist of comparing the results for each item of the questionnaire in the different scenarios in the presence and absence of FFF. The second analysis will compare performance across scenarios and conditions to the Control condition (Flat scenario).

Finally, to examine whether specific scenarios induce different user experiences when using FFF, a descriptive comparison of questionnaire items in each scenario will be made with those from participants in the Control condition performing the Flat scenario.

## 4. Results

A total of 7 participants were tested using the exoskeleton with and without the Foot-to-Forearm Feedback. Of these, six were tested in all five different scenarios (Flat, M-Shape, V-Shape, A-Shape, and Random). One participant (S5) was tested solely in the Flat and the A-Shape scenarios. Lastly, one participant (S4) did not answer the questionnaire regarding the A-Shape scenario. Due to the small number of participants studied, the following section provides mainly a quantitative description of the main objective measures evaluated (number of steps, SIJ angle, SIJ segment length), followed by a qualitative description of the general tendencies found in the data.

### 4.1. Number of Steps

Step counts did not show a consistent trend across scenarios (Table 2), reflecting substantial variability in participants’ gait despite a pre-programmed 1 Hz pattern. This variability was mainly due to differences in foot placement control and leg length. The exoskeleton’s inclination and rotation allowed the modulation of step length, with shorter participants generally taking smaller steps. Technical problems (e.g., loose cables) affected three runs—Participant 2 Random Control, Participant 3 V-Shape FFF, and Participant 7 M-Shape FFF (bolded in Table 2). Overall, these results highlight the interplay between exoskeleton settings and individual participant characteristics.

### 4.2. Brief Interviews

Results from the brief interviews (together with the results from the user experience questionnaire) provided an overall notion of the participants’ reaction to using the exoskeleton, FFF, and the different scenarios. Of the participants analyzed, 5/7 = 71.43% preferred controlling the exoskeleton with the FFF, 1/7 = 14.29% reported no preference between the two conditions, and 1/7 = 14.29% preferred controlling the exoskeleton without the FFF.

### 4.3. User Experience Questionnaire and Interviews

**Figure 4:**
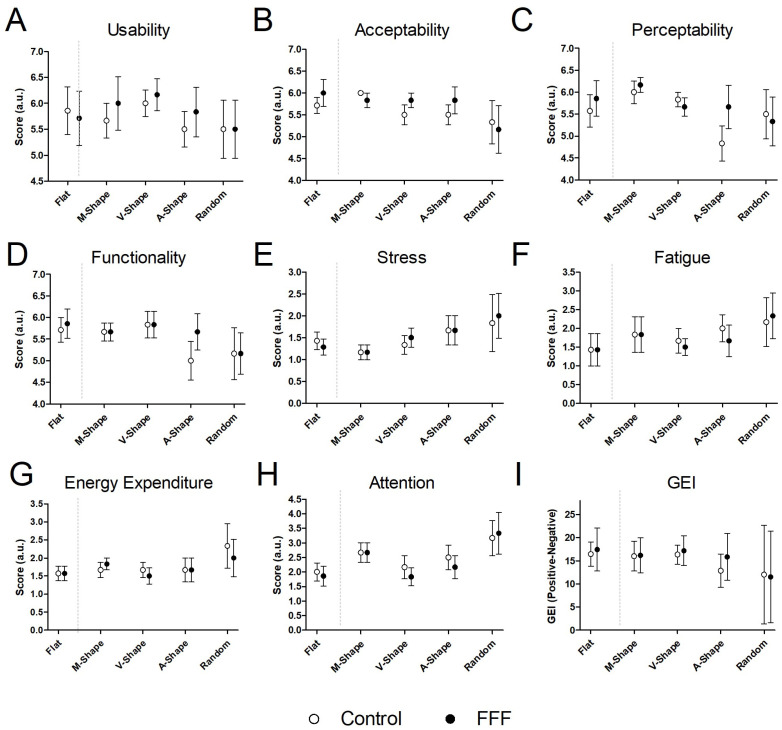
User experience questionnaire. (**A**–**H**) Results for each of the eight items. (**I**) Global user Experience Index (GEI). Mean and Standard Error of the Mean (SEM) are presented. The large SEM in the Random scenario is due to an outlier (Participant S1) in both the Control and FFF conditions.

As indicated in the methods, the following description of the global experience results will not include statistical testing due to the small number of participants. Analysis of the user experience questionnaire suggested trends in differences between items, between scenarios, and between the Control and FFF conditions. Such difference tendencies become more apparent when analyzing maxima, minima, and variability (measured as SEM) to identify patterns occurring throughout the different scenarios or experimental conditions. A summary of the main differences and values for each condition is presented in Figure 4, while the individual responses to each questionnaire item are provided in the Supplementary Information. The largest differences in the questionnaire item Acceptability (Figure 4B) were observed between the Control and FFF conditions in the M-Shape, the V-Shape, and the A-Shape scenarios. Similarly, for Perceptibility (Figure 4C), a large difference was observed between the M-Shape and the A-Shape scenarios. Also noteworthy is the difference in the item Attention (Figure 4H), where both the Control and the FFF conditions showed lower values for the Flat scenario when compared to the Random scenario. Two additional examples of noteworthy differences in Figure 4 relate to the size of the SEM and the effect of the FFF.

In regard to SEM, it appears to be more associated with specific scenarios and with specific items. For example, for the item Usability (Figure 4A), scenarios presented more similar values of SEM, while in Perceptability, Functionality, Stress, and Energy expenditure 281 larger differences were observed. The largest values of SEM were found in the GEI, that is, the index that results from the sum and subtraction of all items (Figure 4I), namely for the Random scenario. These were due to Subject S1 responses after the Random scenario (both in Control and FFF conditions). According to the individual’s report, this scenario was considered particularly difficult due to a previous unpleasant experience with exoskeleton failure in a somewhat similar scenario in the past.

Potentially relevant examples of the effect of the FFF were associated with the items Usability, Perceptibility, and Functionality in the A-Shape scenario (Figure 4A,C,D), where the largest differences between the mean of the Control and the FFF conditions were found.

Lastly, comparison of the changes induced in the longitudinal or transverse axis of the platform suggests that the A-Shape and the Random scenarios (i.e., increases in the longitudinal axis) presented a tendency towards reduced user experience, especially in Perceptibility, Functionality, and Stress (Figure 4C–E). That is, A-Shape and Random scenarios seem to be associated with a tendency toward decreased user experiences for several measures, while Flat, M-Shape, and V-Shape (longitudinal decreases, or transverse increases and decreases) seem to be associated with a tendency toward increased user experiences.

### 4.4. Sacroiliac Joint Length and Angle

**Figure 5:**
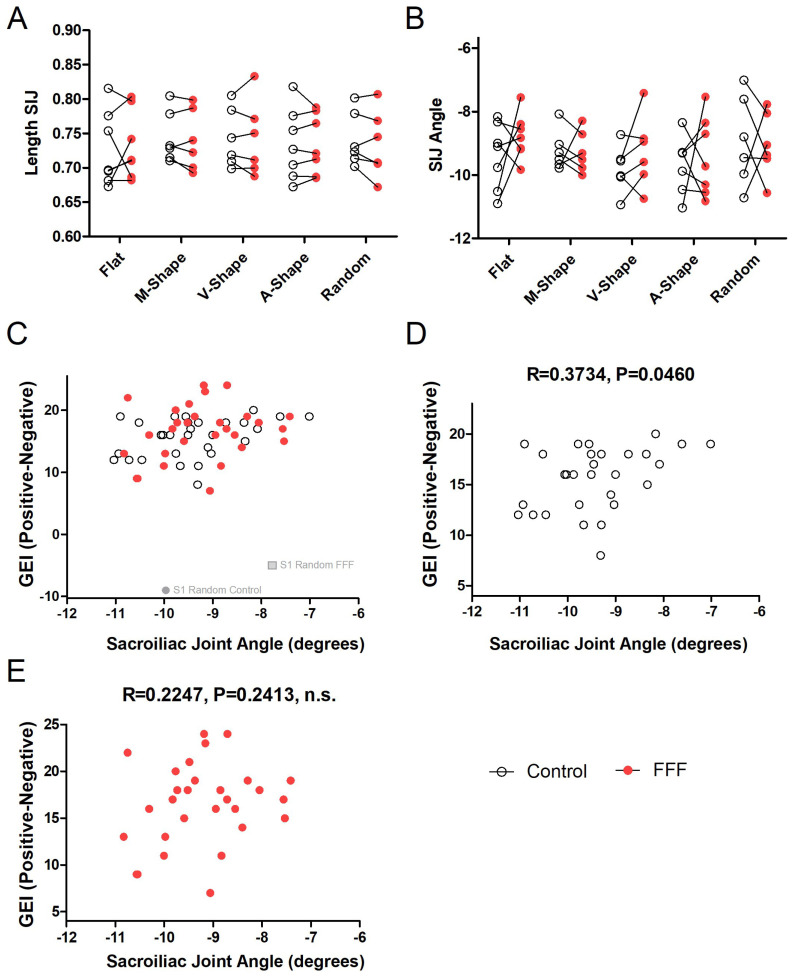
Sacroiliac joint angle and Global Experience Index. (**A**) Segment length between the two sacroiliac joints under Control and FFF conditions across all scenarios. (**B**) SIJ segment angle under Control and FFF conditions, including data from the M-Shape scenario. (**C**) Pooled data from Control and FFF conditions showing the relationship between the Global Experience Index (GEI) with the sacroiliac joint segment (SIJ) angle. Participant S1 was an outlier and was excluded from the subsequent analysis (see text for details). (**D**) Correlation between GEI and SIJ angle in the Control condition (R = 0.3734; *p* = 0.0460). (**E**) Correlation between GEI and SIJ angle in the FFF condition (R = 0.2247; *p* = 0.2413, n.s.).

The distance between the right and left sacroiliac joints was compared across Control and FFF conditions and among the different scenarios. No clear differences were observed in either case. As shown in Panel A of Figure 5, no consistent changes—increases or decreases—were detected across scenarios or conditions for SIJ distance. Similarly, comparison of the SIJ segment angle did not reveal any clear difference between the Control and FFF conditions. Additionally, comparison across scenarios indicated a smaller overall dispersion of values in the M-Shape scenario (Figure 5B).

To determine if the subjective reports were associated with the objective measures gathered from the video recordings, a Pearson correlation between the GEI and the SIJ angle was calculated for the Control and the FFF conditions. The SIJ angle was selected because it presented the largest differences between Control and FFF conditions, as well as between scenarios. The GEI was used because it consisted of an index that reflected the subjective experience resulting from the eight items of the questionnaire.

After excluding Participant S1 in the Random scenario as an outlier (Figure 5C), Pearson correlations were computed separately for Control and FFF conditions. As shown in Figure 5C,D, a reduction in the amplitude of the angle between the right and left sacroiliac joints was modestly correlated with the GEI in the Control condition (R = 0.3734; *p* = 0.046), explaining approximately 14% of the variance in subjective reports (R2 = 0.1394) and indicating that higher GEI scores were associated with larger SIJ angles. By contrast, the correlation was weaker and not significant in the FFF condition (R = 0.2247; R2 = 0.0505; *p* = 0.2413, n.s.).

## 5. Discussion

This study assessed user experience in a small sample of participants controlling an exoskeleton with and without Foot-to-Forearm Feedback (FFF) across five scenarios. The different scenarios included changes in the longitudinal or transverse axis, generating different levels of difficulty. User experience was analyzed through a brief interview as well as through a user experience questionnaire.

Results showed no clear differences, with or without the use of FFF, in the number of steps, the length of the segment between the two sacroiliac joints, or the angle of the sacroiliac joints. Also, no clear differences in the objective measures were observed for the different scenarios, with the potential exception of the M-Shape scenario, where an overall tendency towards a reduction in variability of the data was observed.

Responses to the brief interview revealed that 5/7 participants preferred using the exoskeleton with the Foot-to-Forearm Feedback. For one participant, the use of the FFF did not affect user experience. Only in one case (1/7) did FFF impair the user experience. Meanwhile, comparing the items in the questionnaire suggested that increasing the longitudinal axis (i.e., A-Shape or Random) was associated with an overall decrease in user experience, while a decrease in the longitudinal axis (V-Shape) or an increase in the transverse axis followed by a decrease (M-Shape) resulted in little or no impairment of user experience.

In sum, our results indicate that FFF had no clear detrimental effects across terrains, and most participants preferred using the exoskeleton with FFF. Only one participant reported a less favorable experience, suggesting that while FFF may not benefit every user, it generally does not impede exoskeleton use across different terrains.

Although the sample size was small, these preliminary results highlight the need for further investigation into Foot-to-Forearm Feedback and its potential impact on exoskeleton control in healthy participants. Responses varied across participants and scenarios, suggesting that the effects of FFF may depend on terrain characteristics and individual differences.

### 5.1. Preference for the FFF

At the end of the experiments, users were asked about their preference for the Control or the FFF condition. The majority reported preferring the FFF condition, although the small sample size limits the generalizability of this finding.

Previous work has reported that spinal cord injury patients tend to prefer discrete vibrotactile feedback over continuous or visual/auditory modalities [19]. In other studies, using a brain–machine interface to control an avatar in virtual reality, SCI patients reported that continuous FFF was associated with increased user experience [6,7]. Our results are partially aligned with these reports, showing an overall preference for FFF among healthy participants. However, it is important to note that we tested only healthy participants and used continuous feedback, unlike the discrete feedback in [6,7,19]. Thus, while these observations suggest that FFF is generally well tolerated and may be preferred by participants, the present study cannot provide strong conclusions regarding the relative advantages of continuous versus discrete feedback. Most importantly, FFF did not signifi- cantly impair participants’ performance across the different scenarios, indicating that it can be applied without obvious detriment to control.

### 5.2. User Experience Questionnaires

Analysis of the user experience questionnaire suggests possible differences between scenarios and between the Control and FFF conditions. Most notably, the use of the FFF was associated with the largest differences in the A-Shape scenario for the items Functionality and Perceptibility.

Also, the increases and decreases along the longitudinal and transverse axes suggested that A-Shape and Random scenarios may be associated with an overall reduced user experience, while Flat, V-Shape, and M-Shape scenarios may be associated with increased user experience.

### 5.3. Objective Measures

The objective performance measures evaluated here (i.e., the number of steps, the length of the segment between the sacroiliac joints, and the sacroiliac joint angle) did not reveal any clear differences between the Control and FFF conditions. However, a slight reduction in sacroiliac angle variability was observed in the M-Shape scenario. Notably, the graphs reporting the questionnaire results (where Acceptability, Perceptibility, Fatigue, Energy Expenditure, and Attention were evaluated) showed overall trends consistent with this reduction. It remains unclear whether these trends reflect a simultaneous shift toward increased cognitive and physical engagement while being associated with the lowest levels of stress. In other words, the preliminary results suggest a need to investigate whether the M-Shape scenario represents an optimal balance of intellectual and physical challenge that does not induce stress in participants.

Consistent with the mechanical constraints imposed by the exoskeleton, our analysis showed no consistent differences in SIJ angle between the Control and FFF conditions. This finding could be due to the limited inherent mobility of the joint within the rigid pelvic brace. While the reported kinematic measurements must be treated with caution due to occlusion and soft tissue artifacts resulting from the exoskeleton’s structure, the observed tendency toward reduced SIJ angle variability in the M-Shape scenario (Figure 5B) is noteworthy. This may cautiously suggest that the FFF could be contributing to greater trunk–pelvic stability or improved kinematic coupling within the constrained system, a finding that merits further investigation using specialized motion capture systems in future work.

Lastly, the analysis of the sacroiliac joint segment angle revealed a modest but significant positive correlation under Control conditions. These preliminary findings suggest a potential association between sacroiliac joint kinematics and subjective experience during Control trials, which was not observed under the FFF condition. Given the small sample size, these results should be interpreted descriptively and warrant further investigation. These findings also support the notion that objective measures may be capable of capturing aspects of users’ subjective reports, suggesting potential for their future use [13]. However, it remains unclear why or how this relationship was disrupted under the FFF condition. In previous studies, we reported that an SCI patient controlling a brain–machine interface (BMI) with an FFF associated with an avatar walking found the FFF helpful. Specifically, it allowed him to use the BMI without looking at the floor to determine when the avatar’s foot made contact with the ground [6,7]. Thus, one possibility is that, for participants who preferred using the FFF, the device may have reduced the amount of time they spent looking at the floor to determine foot placement (the video recording resolution used here did not allow exploring this possibility). This change in behavior could have led to fewer postural adjustments, such as changes in the sacroiliac joint angle. This will be the object of future studies.

### 5.4. Potential Neurophysiological Mechanisms and Embodiment

The predominant preference for FFF observed in this study may be interpreted within the framework of multisensory integration, somatosensory plasticity, and embodiment. Cross-modal paired associative stimulation (PAS) protocols demonstrate that temporally precise pairing of tactile stimuli across different body sites can induce Hebbian-like plasticity in somatosensory cortices, even when the afferent input is spatially remapped [19]. Furthermore, recent reviews of PAS targeting frontal and parietal networks show that such protocols modulate functional connectivity and cortical excitability in sensorimotor areas, suggesting that tactile information delivered through non-canonical pathways (e.g., forearm instead of foot) can nevertheless be incorporated into the body schema, thereby reducing reliance on visual feedback and cognitive load [20]. Consistent with this view, non-invasive brain stimulation studies of multisensory integration indicate that embodiment is strengthened when artificial tactile feedback is both temporally and spatially congruent with motor actions [21]. These neurophysiological mechanisms may explain why, during FFF conditions, the positive correlation between sacroiliac joint angle and the Global Experience Index (GEI) observed in the Control condition was not reproduced. A plausible interpretation is that FFF promotes a reorganization of sensorimotor processing in which tactile–proprioceptive coupling predominates, reducing the need for overt postural adjustments and thereby uncoupling subjective experience from the specific kinematic parameter. Such reallocation of sensory processing supports the hypothesis that the reported benefits of FFF—greater comfort and lower perceived effort—stem not merely from physical comfort but from functional plasticity and multisensory integration that precede or modulate objective gait adaptations.

### 5.5. Caveats and Limitations

The small sample size limits the generalizability of our results. However, testing each participant across five scenarios strengthens confidence in the observed patterns.

In addition, the questionnaire applied was based on measurements made in the context of a previous project [13], but it has not been validated. This could potentially lead to bias in the study conclusions. However, the brief interviews conducted after testing provided additional information regarding participants’ experiences. Therefore, the results cannot be extrapolated to the general population due to the small sample size.

Nevertheless, despite the small sample size and the brief questionnaire, it was possible to identify tendencies associated with the use of the FFF and with the different scenarios that support upcoming studies with larger samples and additional scenario complexities. Future studies should complement the self-reported measures with objective performance indicators that quantify the effects of FFF use [6,7,13,14,19,20,21], such as assessments of Fatigue and Energy Expenditure using electromyography or respiratory monitoring devices like spirometers. These additional measures would allow a more comprehensive evaluation of the physiological impact of Foot-to-Forearm Feedback during exoskeleton use.

Lastly, the present study seems to open additional avenues of research in the context of exoskeleton development. First, we have manipulated not only the tactile feedback to the forearm, but we have also tested the effects of the different scenarios (i.e., manipulating the transverse and longitudinal axis of the platform). The preliminary results from the user experience questionnaire and the brief interviews suggest that the M-Shape scenario may provide an optimal balance between difficulty and challenge, making it a promising direction for future research on exoskeleton user control experience.

## 6. Conclusions

The self-reported measures collected in this pilot study suggest that forearm tactile feedback may influence the subjective experience of lower-limb exoskeleton control in healthy participants, but it did not systematically affect the objective measures evaluated here. While most participants expressed a preference for using Foot-to-Forearm Feedback, one reported it as a source of additional stress, highlighting the need for careful consideration in its implementation. These findings highlight how feedback type and terrain geometry jointly shape user perception.

From an application standpoint, FFF provides a simple and low-cost method to deliver multimodal feedback that could be integrated into neurorehabilitation protocols to reinforce sensorimotor learning and embodiment. Future studies should involve larger and clinical samples, employ validated questionnaires, and include physiological measures of effort and fatigue. Overall, FFF appears to be a promising addition to wearable robotic systems, with potential relevance for optimizing exoskeleton-assisted rehabilitation.

## Figures and Tables

**Table 1:** Global experience questionnaire.

Item	Type of Experience Assessed	Definition
Usability	Positive experience item	The degree to which the participant perceived the exoskeleton to be easy to use, with and without the FFF.
Acceptability	Positive experience item	The degree to which the participant perceived the exoskeleton, with and without the FFF, as acceptable for regular use.
Perceptibility	Positive experience item	The degree to which the participant immediately understood the use of the exoskeleton, with and without the FFF.
Functionality	Positive experience item	The degree to which the participant perceived the exoskeleton to be functional, with and without the FFF.
Stress	Negative experience item	The amount of stress perceived by the participant when using the exoskeleton, with and without the FFF.
Fatigue	Negative experience item	The amount of fatigue experienced by the participant from using the exoskeleton, with and without the FFF. This measure referred primarily to a subjective sense of tiredness (e.g., mental fatigue).
Energy expenditure	Negative experience item	The amount of energy the participant perceived to be required for using the exoskeleton, with and without the FFF. This item focused more on the physical demands of the task (e.g., temporary bursts of effort), distinguishing it from fatigue.
Attention	Negative experience item	The amount of attention the participant perceived as required to complete the task, with and without the FFF.
Global Experience Index (GEI)	Composite index	A composite score calculated as the sum of the four positive items minus the sum of the four negative items, ranging from 24 (worst user experience) to 24 (best user experience).

**Table 2:** Number of steps in the Control and FFF conditions for different scenarios across participants; bold numbers indicate runs with technical problems. Except for Participant 5, each participant completed 10 runs (two per scenario).

Participant	Flat		M-Shape		V-Shape		A-Shape		Random	
	Ctrl	FFF	Ctrl	FFF	Ctrl	FFF	Ctrl	FFF	Ctrl	FFF
1	12	12	12	12	13	12	12	11	13	13
2	14	18	17	15	16	20	14	17	**18**	17
3	27	23	26	26	26	**32**	26	22	27	26
4	21	24	23	22	19	21	21	21	19	20
5	22	22	N/A	N/A	N/A	N/A	21	20	N/A	N/A
6	18	21	20	15	19	18	17	18	16	16
7	14	14	14	**17**	15	19	14	15	15	16

Abbreviations: N/A indicates scenarios in which participant 5 was not tested; Ctrl—Control condition.

## Data Availability

The data supporting this study are either in the manuscript or openly available as Suplemmentary Information. The video recordings containing identifiable participant information are not publicly available due to privacy and ethical restrictions.

## Supplementary Materials

Supplementary Data 1 containing the User Experience Questionnaire individual responses can be found in: https://zenodo.org/uploads/17621421#:~:text=10.5281/zenodo.17621421, accessed on 25 October 2025.

## Abbreviations

The following abbreviations are used in this manuscript:

BCI	Brain–Computer Interface
BMI	Brain–Machine Interface
Ctrl	Control
°C	Degrees Celsius
FFF	Foot-to-Forearm Feedback
GEI	Global User Experience Index
Hz	Hertz
IR	Infrared
Kg	Kilogram
LiDAR	Light Detection and Ranging
M	Meter
Mm	Millimeter
N/A	Non-Applicable (not tested)
PAS	Paired Associative Stimulation
RGB	Red Green Blue
SCI	Spinal Cord Injury
SEM	Standard Error of the Mean
SIJ	Sacroiliac joint
USB	Universal Serial Bus
VR	Virtual Reality
